# Interval Timing Deficits Assessed by Time Reproduction Dual Tasks as Cognitive Endophenotypes for Attention-Deficit/Hyperactivity Disorder

**DOI:** 10.1371/journal.pone.0127157

**Published:** 2015-05-18

**Authors:** Shoou-Lian Hwang-Gu, Susan Shur-Fen Gau

**Affiliations:** 1 Graduate Institute of Behavioral Sciences, College of Medicine, Chang Gung University, Tao-Yuan, Taiwan; 2 Department of Psychiatry, National Taiwan University Hospital and College of Medicine, Taipei, Taiwan; 3 Department of Psychology, Graduate Institute of Brain and Mind Sciences, and Graduate Institute of Epidemiology and Preventive Medicine, National Taiwan University, Taipei, Taiwan; Monash University, AUSTRALIA

## Abstract

The literature has suggested timing processing as a potential endophenotype for attention deficit/hyperactivity disorder (ADHD); however, whether the subjective internal clock speed presented by verbal estimation and limited attention capacity presented by time reproduction could be endophenotypes for ADHD is still unknown. We assessed 223 youths with DSM-IV ADHD (age range: 10-17 years), 105 unaffected siblings, and 84 typically developing (TD) youths using psychiatric interviews, intelligence tests, verbal estimation and time reproduction tasks (single task and simple and difficult dual tasks) at 5-second, 12-second, and 17-second intervals. We found that youths with ADHD tended to overestimate time in verbal estimation more than their unaffected siblings and TD youths, implying that fast subjective internal clock speed might be a characteristic of ADHD, rather than an endophenotype for ADHD. Youths with ADHD and their unaffected siblings were less precise in time reproduction dual tasks than TD youths. The magnitude of estimated errors in time reproduction was greater in youths with ADHD and their unaffected siblings than in TD youths, with an increased time interval at the 17-second interval and with increased task demands on both simple and difficult dual tasks versus the single task. Increased impaired time reproduction in dual tasks with increased intervals and task demands were shown in youths with ADHD and their unaffected siblings, suggesting that time reproduction deficits explained by limited attention capacity might be a useful endophenotype of ADHD.

## Introduction

Attention-deficit/hyperactivity disorder (ADHD) has been recognized as an early-onset, common neuropsychiatric disorder with deficits in a wide-range of neuropsychological functions [1]. Despite its high heritability, results regarding the genetic etiologies for ADHD have been mixed [2] due to the tremendous clinical and genetic heterogeneity in ADHD [1, 3, 4]. Hence, the endophenotypic approach has been proposed as one of reasonable methods to reduce the complexity of the phenotypical and potentially genetic heterogeneity and to offer measurable components that are more proximal to the biological expression of genes than the observed behavioral phenotype [4].

A useful endophenotype meets the following criteria [4]: First, the heritable traits of vulnerability are related to the diagnosis; second, these traits can be measured by tools with good psychometric properties; third, the endophenotype shows familial-genetic overlap and increased expression in unaffected relatives of probands [5], such as unaffected siblings [6, 7].

Many studies have shown that several neuropsychological functions [8] including time perception could be the endophenotypes associated with ADHD [5, 9, 10]. Some studies found that ADHD individuals showed poor performance on a variety of time perception tasks [11–15]. Furthermore, some studies with siblings demonstrated that ADHD probands and their unaffected siblings tended to perform poorly in time discrimination tasks of short duration (50 milliseconds) [10], time reproduction tasks of long duration (4–20 seconds) [9], and motor timing of one-second duration [5]. These studies suggested that timing processing can be treated as potential endophenotypes for ADHD [5, 9, 10]; however, different time perception tasks with different temporal information processes might involve different neural substrates [15, 16].

In time discrimination, participants usually need to compare two brief intervals of similar duration in milliseconds [14, 15]. Several studies have suggested that this temporal information might be processed mainly at the perceptual level [14], that it was beyond cognitive control [10], and that the cerebellum might be involved in an important neural anatomical position [10, 14]. In verbal estimation, participants need to report the length of the presented event’s duration (usually in seconds) [17]. Several studies of verbal estimation have dealt with the relationship of subjective time in conventional clock units as a reflection of internal clock speed [18–20]. If the judgments were more/fewer than the presented event’s duration, that is overestimation/underestimation, then the participant might have a faster/slower subjective internal clock than the external objective clock. Time reproduction is a measurement of objective interval timing in which participants need to store and represent a temporal event (not having been told its length) in some way (e.g. press a response key) [12]. Interval timing requires higher cognitive processing [15], especially attention allocation and working memory [21] in order to encode, store, and represent the temporal information, and the interval length increased, the more cognitive demand need to be taxed. Both verbal estimation and time reproduction in seconds require higher cognitive function in order to process the temporal information, and the prefrontal cortex may be involved [22].

One previous study found that children with ADHD and their unaffected siblings were impaired in time reproduction tasks (at 4–20s), and suggested that time reproduction deficits in seconds may be an endophenotype for ADHD [9]. A limited attention capacity and defects in attention allocation are critical processing factors for the deficits in time reproduction in ADHD; however, to the best of our knowledge, no study has examined whether such a limited attention resource would be a candidate endophenotype for ADHD. In addition, no previous study has questioned if verbal estimation of subjective internal clock speed could be a candidate endophenotype for ADHD. The current study used verbal estimation and a time reproduction dual task paradigm to examine this issue.

Some studies have shown that individuals with ADHD had a fast subjective internal clock speed [15, 23], as they usually overestimated the length of intervals in verbal estimation [15, 16]. On the other hand, several neuropsychological models for ADHD have offered different explanations for the time reproduction deficits [15]. Some studies claimed that poor behavioral inhibition impaired working memory and consequently affected the accuracy of interval timing [11, 24]. The delay aversion model claimed that ADHD individuals tended to underestimate durations because of their aversion to task engagement [25]. Attention allocation has been considered to be the critical cognitive process in interval timing [26, 27], and estimated duration is closely related to the amount of attention resources allocated to the passage of time [26], as demonstrated in dual task paradigms [28].

In a time perception dual task paradigm, as participants perform simultaneous temporal and non-temporal tasks, different kinds of information may compete for the limited attention resources [27, 28]. It would be anticipated that the more the attention resources were allocated to the non-temporal task, fewer resources would be available for temporal processing [27, 28]. Consequently, the duration of time passage would be estimated to be shorter and more variable [26, 27]. Hwang et al. [21] used the dual-task paradigm, including time reproduction and non-temporal tasks at time intervals of 5–17 sec and found that children and adolescents with ADHD had less precise time reproduction than did controls across all tasks. They also had a significant increase in differential estimated errors as the interval lengths and task difficulties increased [21]. Our prior study provides some evidence to support the idea that limited attention capacity may account for the deficits in time reproduction in ADHD [21].

Rommelse et al. [9] found that children with ADHD and their unaffected siblings were impaired in time reproduction tasks (at 4–20s), with more absolute discrepancy errors than controls as the length of time increased. They suggested that time reproduction deficits in seconds may be an endophenotype for ADHD; however, no previous study has examined limited attention capacity for time reproduction as a candidate endophenotype for ADHD, and no previous study has examined verbal estimation of internal clock speed as another potential candidate endophenotype for ADHD.

The current study used verbal estimation to explore subjective internal clock speed [19, 20] and a time reproduction dual task paradigm to explore attention allocation in time reproduction [21] to test the following hypotheses: 1) If fast subjective internal clock speed is an endophenotype for ADHD, then ADHD probands and their unaffected siblings will tend to overestimate verbal estimation more than controls; 2) If limited attention capacity, the pathological processing of time reproduction in ADHD, is a proxy for the endophenotype for ADHD, then unaffected siblings, similar to youths with ADHD, will perform worse than controls, especially when the time interval is lengthened and task demands increase because they have fewer attention resources for temporal information processing [21].

## Methods

### Participants

The sample consisted of 223 youths with ADHD (males, 86.9%), 105 unaffected siblings (males, 30.5%), and 84 typically developing (TD) youths (males, 81.0%). The youths with a clinical diagnosis of ADHD in childhood according to DSM-IV criteria were recruited from the Department of Psychiatry, National Taiwan University Hospital, Taiwan. The clinical diagnosis of ADHD was confirmed using the Chinese-language Kiddie epidemiologic version of the Schedule for Affective Disorders and Schizophrenia (K-SADS-E) interview [29] of the participants and their parents at the mean age of 12.3 ±1.6 (range, 10 to 17). Among the 223 youths with a childhood diagnosis of DSM-IV ADHD at the time if the current assessment, 189 (84.8%) still met the full DSM-IV ADHD criteria and 34 (15.2%) did not have overt functional impairment.

We recruited 141 biological siblings, aged 10 or older, who were assessed for their lifetime and current diagnosis of ADHD and other psychiatric disorders using the Chinese K-SADS-E interview at a mean age of 12.7 ±2.4. The inclusion criteria for the unaffected sibling group included the participants who were the biological siblings of youths with ADHD, who did not have lifetime or current diagnosis of ADHD according to the DSM-IV ADHD diagnostic criteria as assessed by the Chinese K-SADS-E interview with the participants and their parents/caregivers, who were aged 8–18, and who and whose parents provided written informed consent to the study. For those families with two or more unaffected siblings, the siblings who have the closest age to the probands would be recruited. Of the 141 biological siblings, 36 (25.5%) were diagnosed either as ADHD or sub-threshold ADHD (more than three of nine symptoms in each of the DSM-IV inattention and hyperactivity-impulsivity dimensions) and 105 who did not have lifetime and current diagnosis of ADHD were identified as unaffected siblings.

The TD youths were recruited from the same school districts as the youths with ADHD with the help of principals and teachers rather than by advertisement. They did not have lifetime or current ADHD based on the Chinese K-SADS-E interviews with them and their parents at their mean age of 12.7±1.9 (age range, 8–18).

All children who had a lifetime clinical diagnosis of psychosis, autism spectrum disorder, or a learning disability or who currently had mood disorders, or who had an intelligence quotient score less than 80 were excluded.

### Measures

#### The Chinese K-SADS-E

The Chinese K-SADS-E was prepared using a two-stage translation and modification of several items with psycholinguistic equivalents relevant to the Taiwanese culture, and further modified to meet the DSM-IV diagnostic criteria [29]. It has been widely used in a variety of clinical [6, 30] and community studies [29] in Taiwan. The details of the interview training and best estimate of psychiatric diagnosis have been described elsewhere [6, 31] and are summarized below.

**Interviewer training:** Four interviewers, who had undergone one year of intensive clinical and research training in child psychiatry before the Chinese K-SADS-E interview training, reached over 90% agreement on all mental disorders assessed by the Chinese K-SADS-E (ranging from 98.25±1.91 to 99.38±1.06) against the rating of each item in the K-SADS-E by SS Gau for 30 clinical subjects before study implementation. The inter-rater reliability of the Chinese K-SADS-E among SS Gau and the four interviewers using 12 subjects was satisfactory for all mental disorders, with generalized kappa for each diagnosis ranging from 0.86 to 1.00. Their K-SADS-E interviews were audiotaped periodically and monitored by SS Gau, who was blind to the personal information of the participants, to ensure the quality of interviews.

**Best Estimate Diagnoses:** The corresponding author was blind to the diagnostic status and name of the participant and was not involved in the direct K-SADS-E interviewing of any of the participants or their parents at follow-up. She made all the best estimates of each psychiatric diagnosis according to the data from the K-SADS-E interviews of participants and their mothers, medical records, and other self-administered questionnaires reported by the participants, parents, and teachers. The diagnostic coding was categorized into definite (meeting all DSM-IV diagnostic criteria), probable (either not meeting all DSM-IV symptoms criteria but more than half or no functional impairment), possible (some symptoms but no impairment), and no diagnosis. For mental disorders other than ADHD, those patients who received a rating as definite or probable by best estimate were categorized as having a particular mental disorder.

#### Chinese version of the Conners’ Parent Rating Scale-Revised: Short Form (CPRS-R:S)

The CPRS-R:S, a 27-item parent-reported rating scale, consists of three factor-derived subscales (cognitive problems/inattentive, hyperactivity, and oppositional) and the ADHD index [32]. Each item is rated on a 4-point Likert scale—0 for never, seldom; 1 for occasionally; 2 for often, quite a bit; and 3 for very often, very frequently. The Chinese CPRS-R:S has been proven to be a reliable and valid instrument for measuring ADHD-related symptoms in Taiwan [32].

#### Time Perception Tasks

Time perception tasks were computerized and controlled by the Visual Basic 6.0 Software Package on an IBM laptop (ThinkPad R51). The joystick made for PS2 was used as the input instrument.

**Verbal estimation Task:** Participants first heard a computer’s sound like /*bi*/ (lasting 100ms from the computer) followed by the presence of a green circle with a diameter of 1.8 cm in the center of a blank screen visible for 5, 12 or 17 seconds. Immediately following each presentation the screen went blank and participants were instructed to verbally estimate the duration of the green circle and respond by a number key on the keyboard. After two practice trials, there were six presentations, two trials for each of three time intervals, 5, 12, and 17 seconds, appearing in random order.

**Time Reproduction Task-Single Version:** The temporal stimulus was identical to that for the verbal estimation (S1 Fig), but immediately following each presentation the screen went blank, and participants were instructed to press the joystick key to let the circle reappear and raise the key when they thought that the same duration of time had elapsed. Following a practice session, two trials, each with the three intervals (5, 12, 17 seconds), were presented in a randomized order for a total of 6 trials.

**Time Reproduction Dual Tasks: Simple and Difficult Versions:** In the dual task, the temporal task was identical to that in the single version as described above. In the concurrent non-temporal task, the participants were asked to count the number of Arabic numerals 1 to 9, 1.5 cm in size, and 1.8 cm under the green circle, with each numeral lasting 200 msec. The inter-stimulus interval (ISI) of the Arabic numerals was randomly distributed, ranging from 1100 msec to 1800 msec in each trial so that the number of appearances of digits in each trial was not the same, even at the same time intervals (S1 Fig). The circle and the numeral stimuli on the screen appeared and disappeared simultaneously. For the timing tasks, the participants were asked “Please try your best to perform the two tasks simultaneously.” Immediately following the temporal and non-temporal stimuli presentation, the screen went blank, and the participants were asked to “Please input the numbers of the target digit by keyboard of the laptop and press the key on the joystick continuously until you think it lasts for the same time interval, then, raise the response key.” Participants were asked to count all the numerals shown on the screen in the simple version of the non-temporal task, and to count only the odd numerals in the difficult version.

**Scoring:** Absolute discrepancy errors and an accuracy coefficient score were the two dependent variables used to measure verbal estimation and time reproduction tasks [11]. An absolute discrepancy error is defined as the absolute value of the magnitude of the discrepancy between the performance on the time perception tasks generated by the participants and the actual time interval presented to them. Because the absolute discrepancy error provides a measure of the magnitude of the errors (incorrectness in time perception tasks) made by the participants, regardless of the direction of the error [11, 12], it is considered as a suitable index to describe the precision of timing performance in the time perception task [11]. An accuracy coefficient score (estimated duration / actual interval) higher than 1.00 indicated overestimation and one lower than 1.00 indicated underestimation. The value of the accuracy coefficient score has been used as an index to reflect the speed of subjective internal clock in some studies [19, 20] and has also been used to describe the direction of the errors of estimation [11]. The accuracy of the non-temporal task was defined as the ratio of the number of conditions in which the subjects had a perfect score for the digits shown on the screen to the total number of conditions.

### Procedure

The Research Ethics Committee of National Taiwan University Hospital [IRB ID: 9561703011; ClinicalTrials.gov number NCT00491647] approved the current study prior to its implementation. Written informed consent was obtained from the participants and parents. All participants received the same psychiatric evaluation and time perception tasks. The participants and their parents, mainly mothers (76.7%), were interviewed using the Chinese K-SADS-E independently by separate well-trained interviewers to determine the diagnosis of ADHD and other psychiatric disorders as described by the DSM-IV diagnostic criteria. The participants were assessed using the Wechsler Intelligence Scale for Children-3rd edition (WISC-III), and only those participants who met the inclusion criteria were assessed with the time reproduction tasks. The participants who took methylphenidate were asked to halt medication for at least 24 hours before the tests.

### Data Analyses

We used SAS 9.2 software (SAS Institute Inc., Cary, NC, USA) to perform the data analysis. The descriptive results were displayed as frequency and percentage for categorical variables, and mean and SD for continuous variables. We used a multi-level model with random and fixed effects to address the lack of independence of youths with ADHD and their unaffected siblings within the same family, the repeated measures at different time intervals, and the different versions of tasks by the same participants. The Proc Glimmix procedure with binomial distribution and logit link for the non-linear mixed model was used to compare the rates of co-morbid psychiatric disorders and to compute the odds ratios and 95% confidence intervals. We excluded the outliers beyond 3 SDs for each condition of the time perception tasks. The Proc Mixed procedure was used with the linear mixed model to address the lack of independence within the same family for probands and unaffected siblings and the repeated measures within the same participants, and to compare each version of the time perception task. The three comparison groups were treated as a fixed factor and the three time interval lengths as repeated measures; the Bonferroni method was used to adjust *p* values in post hoc analyses. Cohen’s d was used to compute the effect size [33].

In order to examine the effect of attention allocation in time reproduction paradigms, a linear mixed model with fixed and random effects was used to address repeated measures for the same participants. The three comparison groups were treated as a fixed factor, the three time interval lengths and three different tasks as repeated measures, and parental educational level and participants’ gender, age, full-scale IQ, use of methylphenidate, presence of psychiatric co-morbidity, and symptoms of ADHD as measured by the CPRS-R:S as covariates in the multivariate analysis. Two-way interaction terms including “groups and interval lengths interaction”, and “groups and different versions of time reproduction task” were also included in the multivariate analysis.

Pearson’s Correlations were performed to explore the associations between the performance of verbal estimation, time reproduction (single, simple/ difficult versions of the dual task), and the symptoms of inattention, hyperactivity and impulsivity as measured by the CPRS-R:S. Exploratory stepwise regression analyses were then performed to determine which symptoms (inattention, hyperactivity and impulsivity) as measured by the CPRS-R:S accounted for the most variability in each time perception task.

## Results

### Sample description

There were significant differences in gender, level of parental educational, inattentive symptoms and hyperactivity-impulsivity symptoms among the three comparison groups (Table 1). One hundred eighty-six youths with ADHD (83.4%) had taken and/or were taking methylphenidate (Table 1). The distribution of ADHD subtypes in the ADHD group was 121 (54.3%) for the ADHD-Combined subtype, 90 (40.4%) for the ADHD-Inattentive subtype, and 12 (5.4%) for the ADHD-Hyperactivity-Impulsivity subtype. Youths with ADHD were more likely than unaffected siblings and TD youths to have oppositional defiant disorder and conduct disorder. The details of the comparison of co-morbid psychiatric disorders among the three groups are provided in S1 Table.

**Table 1:** Sample characteristics.

	ADHD (n = 233)	Unaffected Sibling (n = 105)	TD (84)	*F* value or *χ2*
	mean (SD) or %	mean (SD) or %	mean (SD) or %	
Age	12.3 (1.6)	12.7 (2.4)	12.7 (1.9)	2.61
Gender, male	86.9%	30.5%	81.0%	115.84***
Full IQ	102.6 (11.8)	105.0 (11.7)	112.3 (8.4)	22.62***
Medication (methylphenidate)				
Current use	49.8%		—	
Ever used	83.4%		—	
Duration of use (in months)	13.11 (6.07)			
Father’s Education Level				
College and above	62.7%		87.9%	15.65***
Senior high school and below	37.3%		12.1%	
Mother’s Education Level				
College and above	52.1%		77.6%	13.92***
Senior high school and below	47.9%		22.4%	
CPRS-R: S^a^				
Inattention/Cognitive problems	10.98(4.42)	1.23(1.98)	1.29(1.94)	349.82***
Hyperactivity/Impulsivity	7.88(4.86)	0.69(1.71)	0.47(0.99)	179.09***

**Note.** ADHD = Attention-Deficit/Hyperactivity Disorder, TD = typically developing adolescents, SD = standard deviation,

^a^CPRS-R: S = Conners’ Parent Rating Scale- Revised: Short Form

*** *p* < .001

### Verbal Estimation Task

ADHD probands had more absolute discrepancy errors than unaffected siblings (*t*_(764)_ = 3.78, *p*< .001) and TD youths (*t*_(764)_ = 4.21, *p*< .001), and the significant effects remained after controlling for covariates. The precision decreased with increased interval length, but the interaction effects between the groups and intervals were not significant (Table 2).

**Table 2:** Comparisons of performance on the verbal estimation task among the ADHD, unaffected sibling, and typically developing groups.

mean(SD)	ADHD (n = 223)	Unaffected Sibling (n = 105)	TD (n = 84)	Cohen’s *d*			Multivariate Analysis			
				A vs. S	A vs. TD	S vs. TD	*F* value	Comparison	*F* value^a^	Comparison^a^
Absolute discrepancy errors										
5 sec	2.14(2.38)	1.57(1.76)	1.13(1.30)	0.40	0.67	0.28	G:*F* = 13.52, *p*<.001;	A>S, TD	G:*F* = 5.99, *p* = .003;	A>S, TD
12 sec	4.61(5.00)	3.76(4.25)	2.70(3.33)	0.18	0.45	0.28	IN:*F* = 85.53, *p*<.001;		IN:*F* = 65.79, *p*<.001;	
17 sec	6.54(7.42)	5.57(6.45)	3.58(3.28)	0.14	0.52	0.39	G×IN:*F* = 2.00, *p* = .092		G×IN:*F* = 1.68, *p* = .153	
Accuracy Coefficient Scores										
5 sec	1.38(0.75)	1.15(0.44)	1.06(0.31)	0.37	0.55	0.24	G:*F* = 27.4, *p*<.001;	A >S, TD	G:*F* = 6.71, *p* = .001;	A>S, TD
12 sec	1.28(0.57)	1.15(0.52)	1.04(0.35)	0.24	0.51	0.25	IN:*F* = 1.65, *p* = .193;		IN:*F* = 1.64, *p* = .194;	
17 sec	1.30(0.67)	1.14(0.48)	1.01(0.28)	0.27	0.56	0.33	G×IN:*F* = 0.73, *p* = .573		G×IN:*F* = 0.60, *p* = .663	

**Note.** M, mean; SD, standard deviation; ADHD, attention deficit/hyperactivity disorder; TD, typically developing adolescents; G, group main effects, IN, lengths of interval main effects;

^a^ adjusted for covariates (age, gender, co-morbidity, and parental education)

ADHD probands had higher accuracy coefficient scores than unaffected siblings (*t*_(764)_ = 6.37, *p*< .001) and TD youths (*t*_(764)_ = 4.67, *p*< .001) which implied that they tended to overestimate the length of duration. The significant effects remained after controlling for covariates. The accuracy coefficient scores did not significantly change with increased interval length and no significant interaction effects were found between the groups and intervals.

### Time Reproduction Single Task

ADHD probands had more absolute discrepancy errors than unaffected siblings (*t*_(858)_ = 3.65, *p*< .001) and TD youths (*t*_(858)_ = 2.53, *p* = .035), but the significant effect disappeared after controlling for covariates. The precision decreased with increased interval length, and the interaction effect between the groups and intervals was significant, suggesting a significant difference among the groups with increased interval length (Table 3).

**Table 3:** Comparisons of performance on the time reproduction task among the ADHD, unaffected sibling, and typically developing groups.

M(SD)	ADHD	Unaffected Sibling	TD	Cohen’s d			Multivariate Analyses			
	(n = 223)	(n = 105)	(n = 84)	A vs. S	A vs. TD	S vs. TD	*F* value	Comparison	*F* value ^a^	Comparison ^a^
Absolute discrepancy errors										
Time reproduction (single)										
5 sec	0.75(0.50)	0.78(0.55)	0.73(0.41)	-0.06	0.04	0.10	G: *F* = 8.08, *p* <.001;	A>S, TD	G: *F* = 2.17, *p* = .115;	n.s.
12 sec	1.91(1.53)	1.30(1.11)	1.63(1.50)	0.46	0.18	-0.25	IN:*F* = 92.96, *p*<.001;		IN: *F* = 68.22, *p*<.001;	
17 sec	2.57(2.39)	2.25(2.07)	1.94(2.00)	0.14	0.29	0.15	G×IN:*F* = 2.91, *p* = .021		G×IN:*F* = 3.30, *p* = .011	
Time reproduction (dual task simple version)										
5 sec	1.17(0.78)	1.15(0.66)	0.98(0.58)	0.03	0.28	0.27	G: *F* = 7.24, *p*<.001;	A,S>TD	G: *F* = 5.07, *p* = .007;	A>TD
12 sec	3.08(2.04)	2.89(2.08)	2.45(1.40)	0.09	0.36	0.25	IN:*F* = 293.45, *p*<.001;		IN:*F* = 224.29, *p*<.001;	
17 sec	4.70(2.97)	4.49(2.63)	3.55(2.13)	0.07	0.44	0.39	G×IN: *F* = 2.47, *p* = .043		G×IN:*F* = 3.39, *p* = .009	
Time reproduction (dual task difficult version)										
5 sec	1.48(0.88)	1.44(0.80)	1.16(0.70)	0.05	0.40	0.37	G: *F* = 8.35, *p*<.001;	A,S>TD	G: *F* = 3.69, *p* = .025;	A>TD
12 sec	3.64(2.29)	3.51(2.10)	2.82(1.74)	0.06	0.40	0.36	IN: *F* = 305.51, *p*<.001;		IN: *F* = 239.54, *p*<.001;	
17 sec	5.85(3.57)	5.24(3.16)	4.38(2.95)	0.18	0.45	0.28	G×IN: *F* = 2.76, *p* = .027		G×IN:*F* = 3.09, *p* = .016	
Accuracy coefficient scores										
Time reproduction (single)										
5 sec	0.93(0.24)	0.89(0.14)	0.87(0.10)	0.20	0.33	0.16	G: *F* = 0.93, *p* = .396;	n.s.	G: *F* = 0.93, *p* = .395;	n.s.
12 sec	0.87(0.19)	0.92(0.12)	0.88(0.13)	-0.31	-0.06	-0.32	IN: *F* = 0.26, *p* = .772;		IN: *F* = 0.30, *p* = .740;	
17 sec	0.86(0.20)	0.90(0.14)	0.90(0.12)	-0.23	-0.24	0.00	G×IN: *F* = 3.28, *p* = .011		G×IN: *F* = 2.90, *p* = .021	
Time reproduction (dual task simple version)										
5 sec	0.83(0.29)	0.84(0.21)	0.86(0.16)	-0.04	-0.13	-0.11	G: *F* = 3.30, *p* = .037;	TD>A	G: *F* = 2.42, *p* = .090;	n.s.
12 sec	0.77(0.23)	0.77(0.22)	0.81(0.13)	0.00	-0.21	-0.22	IN: *F* = 20.97, *p*<.001;		IN: *F* = 19.31, *p*<.001;	
17 sec	0.72(0.22)	0.75(0.18)	0.81(0.14)	-0.15	-0.48	-0.37	G×IN: *F* = 0.88, *p* = .474		G×IN:*F* = 1.13, *p* = .340	
Time reproduction (dual task difficult version)										
5 sec	0.78(0.33)	0.76(0.22)	0.82(0.19)	0.07	-0.15	-0.29	G: *F* = 6.03, *p* = .003;	TD>A,S	G: *F* = 2.48, *p* = .084;	n.s.
12 sec	0.71(0.24)	0.71(0.20)	0.80(0.19)	0.00	-0.42	-0.46	IN: *F* = 15.22, *p*<.001;		IN: *F* = 14.36, *p*<.001;	
17 sec	0.66(0.24)	0.69(0.21)	0.76(0.19)	-0.13	-0.46	-0.35	G×IN: *F* = 1.25, *p* = .1.25		G×IN: *F* = 0.80, *p* = .523	

**Note.** M, mean; SD, standard deviation; ADHD, attention deficit/hyperactivity disorder; TD, typically developing adolescents; G, group main effects, IN, lengths of interval main effects;

^a^ adjusted covariates (age, gender, co-morbidity, and parental education)

There were no significant differences in accuracy coefficient scores among the three groups and the intervals, but the effects of interaction between groups and intervals were significant after controlling for covariates (Table 3).

### Time Reproduction Dual Tasks

**Fig 1:**
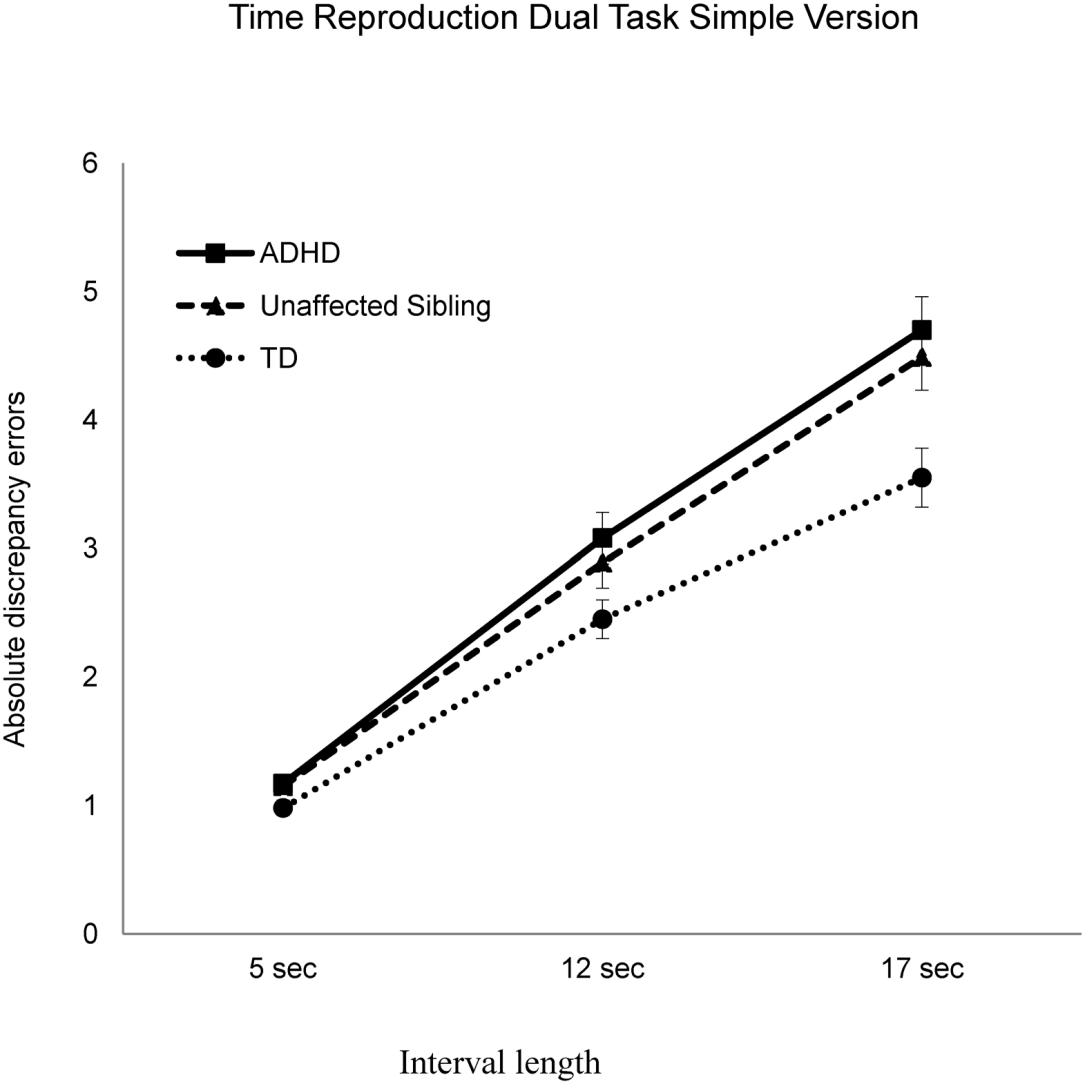
Absolute discrepancy errors on the simple version of the time reproduction dual task in three time interval lengths (5, 12, and 17 seconds) for the attention deficit hyperactivity disorder (ADHD), unaffected siblings, and typically developing adolescents (TD) groups.

**Fig 2:**
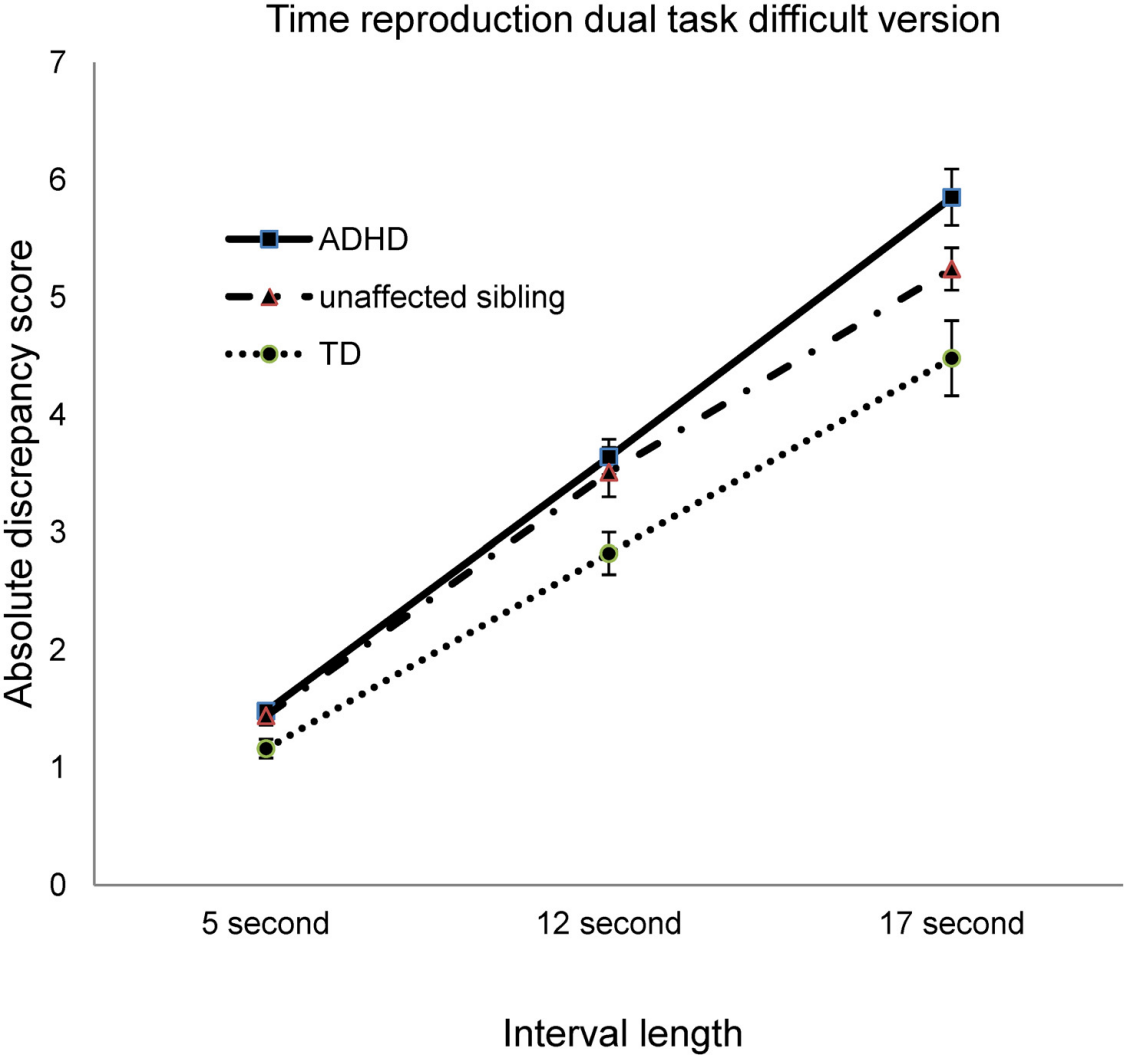
Absolute discrepancy errors on the difficult version of the time reproduction dual task in three time interval lengths (5, 12, and 17 seconds) for the ADHD, unaffected siblings, and TD groups.

ADHD probands (*t*_(778)_ = 3.79, *p*< .001 for the simple version; *t*_(786)_ = 4.06, p< .001 for the difficult version) and unaffected siblings (*t*_(778)_ = 2.58, p = .030 for the simple version; *t*_(786)_ = 2.80, p< .016 for the difficult version) had more absolute discrepancy errors than did TD youths after controlling for accuracy rates in the non-temporal tasks in the two versions of the time reproduction dual tasks, but only the significant difference between ADHD probands and TD youths remained after controlling for covariates in the simple (*t*_(655)_ = 3.18, *p* = .002) and difficult (*t*_(663)_ = 2.68, *p* = .022) versions of the time reproduction dual tasks. The precision decreased with increased interval length and the interaction effect between the three groups and intervals (Table 3) was significant, suggesting a significant difference among the three groups with increased interval lengths in the two versions of time reproduction dual tasks (Figs 1 and 2). Details of the comparison of accuracy in the non-temporal tasks among the three groups are provided in S2 Table.

There were no significant differences in accuracy coefficient scores among the three groups after controlling for covariates. Participants tended to underestimate time as the interval lengths increased without statistically significant groups and time interval interactions (Table 3).

### A model integrating the three interval lengths and three time reproduction task designs in absolute discrepancy errors

In order to compare the interactions between the three groups and the levels of difficulty in the time reproduction tasks (including the three interval lengths and single task and dual tasks in the simple and difficult versions) in absolute discrepancy scores, a mixed model was used to account for intra-family correlations and repeated measures in the same subjects (Table 4). The backward elimination method was used to determine the significant correlates for the precision of time reproduction in the final model. We found that there were significant contrast effects between youths with ADHD and TD youths in interval length (17-seconds vs. 5-seconds, *F*_(1, 2402)_ = 22.48, *p* < .001), and between unaffected siblings and TD youths in interval lengths (17-seconds vs. 5-seconds, *F*_(1, 2402)_ = 6.60, *p* = .010). There were contrast effects between youths with ADHD and TD youths in task design (simple vs. single, *F*_(1, 2402)_ = 5.83, *p* = .016; difficult vs. single, *F*_(1, 2402)_ = 9.84, *p* = .002); and between unaffected siblings and TD youths in task design (simple vs. single, *F*_(1, 2402)_ = 6.22, *p* = .013; difficult vs. single, *F*_(1, 2402)_ = 5.06, *p* = .025). The results showed that increased length of time intervals and task difficulty and complexity were associated with an increased magnitude of estimation errors among youths with ADHD and their unaffected siblings when compared to TD youths, after controlling for all the covariates.

**Table 4:** A model integrating the effects of group, time interval, and task versions on the absolute discrepancy errors on the time reproduction task after controlling for confounding factors.

	β	95% CI	F value	p†
Group Effect				
ADHD vs. TD	-0.62	(-1.11, -0.13)	6.13	.013
Unaffected Sibling vs. TD	-0.42	(-0.96, 0.13)	2.27	.132
Time Interval Effect				
12 sec (vs. 5 sec)	1.38	(1.01, 1.75)	53.68	<.001
17 sec (vs. 5 sec)	2.19	(1.82, 2.56)	134.84	<.001
Task Demand Effect				
Simple (vs. Single)	0.76	(0.39, 1.13)	16.10	<.001
Difficult (vs. Single)	1.24	(0.87, 1.61)	43.02	<.001
2-way Interactions				
ADHD*17 sec (vs. 5 sec)	1.03	(0.61, 1.46)	22.46	<.001
Unaffected Sibling*17 sec (vs. 5 sec)	0.63	(0.15, 1.11)	6.60	.010
ADHD* Simple (vs. Single)	0.53	(0.10, 0.96)	5.76	.016
ADHD* Difficult (vs. Single)	0.68	(0.25, 1.11)	9.69	.002
Unaffected Sibling* Simple (vs. Single)	0.61	(0.13, 1.09)	6.22	.013
Unaffected Sibling* Difficult (vs. Single)	0.55	(0.07, 1.03)	5.06	.025

**Note.** ADHD, Attention-Deficit/Hyperactivity Disorder, TD, typically developing youths, CI, confidence interval; β, regression coefficient estimates

^†^ after controlling for gender, age, Full-scale IQ, co-morbid conditions, the symptoms of inattentive, hyperactivity, and impulsivity as measured by the CPRS-R:S, and parental education levels.

### Dimensional analyses

To explore the association between symptoms of ADHD and verbal estimation and time reproduction, we calculated the Pearson correlations (*r*) between inattention and hyperactivity-impulsivity as measured by CPRS-R:S and the absolute discrepancy errors and accuracy coefficient scores for verbal estimation and the three kinds of time reproduction tasks (S3 Table). There were significantly low positive correlation between inattention symptoms and absolute discrepancy scores in verbal estimation and those in time reproduction (*r* = .23, .26, p < .05, respectively) (S3 Table). Moreover, there were significantly low positive correlation between inattentive symptoms and overestimation in verbal estimation (*r* = .23, p < .05) (S3 Table) and low negative correlations between inattention symptoms and the underestimation in time reproduction in time reproduction dual task difficult version (*r* = -.21, p < .05) (S3 Table).

As shown in S4 Table, in stepwise regressions analyses, only inattention symptoms accounted for 6.1% of the variability in the absolute discrepancy scores in the dual task difficult version as presented by the index of sr^2^ (the squared semipartial correlations).

## Discussion

To the best of our knowledge, the current study was the first to examine verbal estimation and time reproduction performance with different levels of cognitive demands in temporal tasks using additive methods [34] in a large sample of youths with ADHD and their unaffected siblings, as compared to TD youths. We found that ADHD probands tended to overestimate in verbal estimation more than their unaffected siblings and TD youths, suggesting that subjective fast internal clock speed was seen in youths with ADHD, and might be a characteristic of ADHD [5]; however, we found that ADHD probands and their unaffected siblings were less precise in time reproduction dual tasks (simple and difficult versions) in terms of absolute discrepancy errors. As the task demand increased in the time reproduction tasks, differential patterns were shown in the contrasting effects between groups (ADHD vs. TD, unaffected sibling vs. TD) and interval lengths (17 sec vs. 5 sec), and between groups and task difficulty (simple vs. single; difficult vs. single). Unaffected siblings occupied an intermediate position between youths with ADHD and TD youths in both versions of the time reproduction dual tasks. Our results provided strong evidence to support the previous findings that ADHD youths have a limited attention capacity causing the defect inattention allocation involved in time reproduction [21]. The current study also provided new evidence implying that the limited attention capacity revealed in the time reproduction dual tasks in 5–17 seconds fulfilled some of the important criteria for a candidate endophenotype for ADHD[4] because: 1) the time reproduction task had good psychometric properties, especially for the dual task paradigm [21]; 2) time reproduction deficits related to the limited attention capacity and the defect of attention allocation were found in youths with ADHD [21]; 3) time reproduction deficits related to the limited attention capacity were also noted in unaffected siblings of youths with ADHD. In dimensional analyses, the results showed that the severity of inattentive symptoms in ADHD was associated with the magnitude of absolute discrepancy errors on the time reproduction dual task difficult version.

Although impulsive behaviors are essential symptoms of ADHD [23], past studies did not consistently show that ADHD youths had a tendency to overestimate verbal estimations [15]. In the current study with a large sample adjusted for several confounding factors, we found that ADHD youths tended to overestimate and made more errors of estimation in verbal estimations at 5–17 seconds than did unaffected siblings and TD youths. As a result, there is no evidence to support the hypothesis that verbal estimation of time could be considered as a potential endophenotype of ADHD.

Our finding that accuracy coefficients on verbal estimation were not impacted by the length of intervals, but that accuracy coefficients on time reproduction dual tasks decreased as the length of intervals increased suggested that time reproduction might have a different timing process compared to verbal estimation. One possible explanation would be that, in verbal estimation, participants transformed the monitored duration into the subjective timing unit with verbal labels [35]; however, in time reproduction, participants needed to encode, store and represent the temporal information for a longer period of time [35]. As the length of intervals increased, more temporal information needed to be processed by attention resources, and less accuracy was shown by all participants on both versions of the time reproduction dual tasks.

In terms of absolute discrepancy errors, we found that ADHD probands and unaffected sibling performed worse as the length of the interval increased [9]. The time reproduction dual tasks design has demonstrated that attention capacity and allocation may be the critical processes in the precision of time reproduction [21, 27]. Most importantly, in our prior study using two versions of the time reproduction dual task, the more demanding the non-temporal task in which participants needed to count the number of digits, the fewer the attention resources that were allocated to temporal information processing, and the worse the precise performance in time reproduction tasks, especially in probands with ADHD [21] and their unaffected siblings. As attention is needed to decode and to process the information for timing in time reproduction [36], poor performance in time reproduction by ADHD probands and their unaffected siblings may be associated with limited attention capacity [21]. To this end, our finding lends evidence to support the hypothesis that limited attention capacity as assessed by the time reproduction dual task may be a potential cognitive endophenotype for ADHD.

The time reproduction dual task requires great attention capacity [21], and prior studies showed that symptoms of inattention were associated with a deficit in executive function in ADHD, including performance in attention tasks [37]. In line with the findings of prior studies, it is therefore reasonable to assume that performance on the time reproduction dual task would be associated with the severity of inattention symptoms in ADHD as shown in the current study.

### Limitations

There are some major methodological limitations to the current study. First, the clinic-based sample of ADHD probands may introduce selection bias and our findings may not be able to be generalized to community-based samples. Second, the ratio of gender in unaffected siblings was significantly different from the gender ratio in ADHD probands and TD youths. This made generalization of our results questionable; however, gender did not have an effect on the performance on the time perception tasks in all participants (S5 Table), and gender was one of the covariates in our statistical results. This suggested that gender was independent of limited attention capacity in time reproduction as a candidate endophenotype for ADHD. Third, we did not have a specific recruitment strategy for age, gender, or co-morbidity, so that the clinic-based sample of adolescents with ADHD demonstrated clinical heterogeneity. We did carefully include age, gender, co-morbid psychiatric conditions, parental education, and full-scale IQ as covariates in the statistical model and found robust group differences which increased as the levels of task demands increased.

## Conclusions

Several studies have shown ADHD to be associated with deficits in different timing functions in seconds [15], and Rommelse et al. [9] suggested that time reproduction might be a candidate endophenotype for ADHD. The current study clearly provided evidence to support the idea that deficits in attention allocation may offer a suitable explanation for ADHD time reproduction problems, and limited attention capacity as assessed by the time reproduction dual task with different time intervals and levels of difficulty on non-temporal tasks may be a candidate endophenotype for ADHD. A speeded-up internal clock as assessed by verbal estimation of time was associated with the ADHD diagnosis itself as a state marker, rather than a trait marker for this disorder. The clinical implication of our findings is that extra assistance and educational intervention are needed not only for youths with ADHD, but also for their unaffected siblings, particularly when they are assigned to do complex tasks with their limited attention capacity. To better understand the relationship between attention allocation and its underlying mechanisms related to genetic etiology of ADHD, we are planning a study which will integrate genetic, neuropsychological, and neuroimaging data.

## Supporting Information

S1 Fig The diagrams for the temporal stimuli of all the time perception tasks. (DOCX)

S1 Table Psychopathology of youths with ADHD, unaffected siblings and TD youths. (DOC)

S2 Table The Accuracy of the Non-Temporal Tasks of the Time Reproduction Dual Tasks. (DOC)

S3 Table The correlations between ADHD symptoms and time perception tasks in the absolute discrepancy score/in the accuracy coefficient scores. (DOCX)

S4 Table The regression analyses for ADHD symptoms as predictors and time reproduction dual task (difficult) as the dependent variables in the absolute discrepancy score. (DOCX)

S5 Table Gender and interval length effect in verbal estimation and time reproduction tasks. (DOCX)
