# Supplementary material for: Interval Timing Deficits Assessed by Time Reproduction Dual Tasks as Cognitive Endophenotypes for Attention-Deficit/Hyperactivity Disorder
Source: PLoS One. 2015 May 18;10(5):e0127157. doi: 10.1371/journal.pone.0127157 (PMC4436371; doi:10.1371/journal.pone.0127157)
Supplement: S1 Fig — (DOCX) [file pone.0127157.s001.docx]

|  | Verbal estimation | Time reproduction single version | Time reproduction dual task  (simple version) | Time reproduction dual task  (difficult version) |
| --- | --- | --- | --- | --- |
| Temporal stimuli |  |  | 5  5  5  5 | 5  5 |

S1 Fig. The diagrams for the temporal stimuli of all the time perception tasks.
