# Supplementary material for: Interval Timing Deficits Assessed by Time Reproduction Dual Tasks as Cognitive Endophenotypes for Attention-Deficit/Hyperactivity Disorder
Source: PLoS One. 2015 May 18;10(5):e0127157. doi: 10.1371/journal.pone.0127157 (PMC4436371; doi:10.1371/journal.pone.0127157)
Supplement: S1 Table — (DOC) [file pone.0127157.s002.doc]

**S1 Table.** Psychopathology of youths with ADHD, unaffected siblings and TD youths

| Lifetime Psychiatric Diagnoses | ADHD  (N = 223) | | Unaffected Sibling  (N=105) | | TD  (N = 84) | | Odds Ratio (95% CI) or p values | | | | |
| --- | --- | --- | --- | --- | --- | --- | --- | --- | --- | --- | --- |
|  |  |  |  |  |  |  | ADHD vs. TD | Sibling vs. TD | | | ADHD vs. Sibling |
|  | N | (%) | N | (%) | N | (%) |  | |  |  | |
| ADHD-Combined type | 121 | (54.3) |  |  |  |  |  | |  |  | |
| ADHD-Inattentive type | 90 | (40.4) |  |  |  |  |  | |  |  | |
| ADHD-Hyperactivity type | 12 | (5.3) |  |  |  |  |  | |  |  | |
| Oppositional Defiant Disorder | 115 | (52.3) | 9 | (8.6) | 3 | (3.6) | 29.56(8.89-98.50 | | 2.53(0.65-9.89) | 11.69(5.54-24.65) | |
| Conduct Disorder | 33 | (14.9) | 0 | (0) | 0 | (0) | <.001* | | --- | <.001* | |
| Tic Disorder | 11 | (5.0) | 1 | (1.0) | 1 | (1.2) | 4.35(0.54-35.24) | | 0.80(0.05-13.49) | 5.44(0.67-44.00) | |
| Mood Disorders | 15 | (6.8) | 3 | (2.9) | 2 | (2.4) | 2.99(0.65-13.63) | | 1.21(0.19-7.58) | 2.48(0.69-8.90) | |
| Anxiety Disorders | 46 | (20.8) | 14 | (13.3) | 16 | (19.0) | 1.11(0.59-2.13) | | 0.65(0.30-1.45) | 1.71(0.88-3.30) | |
| Any psychiatric disorder | 156 | (70.6) | 28 | (26.7) | 22 | (26.2) | 6.78(3.78-12.17) | | 1.04(0.53-2.04) | 6.52(3.83-11.12) | |

**Note.** ADHD, Attention-Deficit/Hyperactivity Disorder; TD, Typically developing adolescents, *: *P* value of Fisher Exact Test
