# Supplementary material for: Interval Timing Deficits Assessed by Time Reproduction Dual Tasks as Cognitive Endophenotypes for Attention-Deficit/Hyperactivity Disorder
Source: PLoS One. 2015 May 18;10(5):e0127157. doi: 10.1371/journal.pone.0127157 (PMC4436371; doi:10.1371/journal.pone.0127157)
Supplement: S2 Table — (DOC) [file pone.0127157.s003.doc]

**S2 Table.** The Accuracy of the Non-Temporal Tasks of the Time Reproduction Dual Tasks

|  | ADHD (n=223) | Unaffected Siblings  (n=105) | TD  (n=84) | Statistics | |
| --- | --- | --- | --- | --- | --- |
|  | Mean (SD) | Mean (SD) | Mean (SD) | *F* value | *p* value |
| Dual task (simple) |  |  |  |  |  |
| 5 sec | 0.64 (0.35) | 0.70 (0.35) | 0.71 (0.34) | 1.33 | 0.27 |
| 12 sec | 0.36(0.36) | 0.41 (0.37) | 0.32 (0.35) | 1.35 | 0.26 |
| 17 sec | 0.24 (0.32) | 0.27 (0.33) | 0.25 (0.34) | 0.14 | 0.87 |
| Dual task (difficult) |  |  |  |  |  |
| 5 sec | 0.77(0.30) | 0.81 (0.31) | 0.84 (0.31) | 1.39 | 0.25 |
| 12 sec | 0.57 (0.35) | 0.62 (0.38) | 0.64 (0.36) | 1.30 | 0.28 |
| 17 sec | 0.44 (0.37) | 0.54 (0.42) | 0.54 (0.32) | 2.67 | 0.07 |

**Note.** ADHD, Attention-Deficit/Hyperactivity Disorder; TD, typically developing youths
