# Supplementary material for: Interval Timing Deficits Assessed by Time Reproduction Dual Tasks as Cognitive Endophenotypes for Attention-Deficit/Hyperactivity Disorder
Source: PLoS One. 2015 May 18;10(5):e0127157. doi: 10.1371/journal.pone.0127157 (PMC4436371; doi:10.1371/journal.pone.0127157)
Supplement: S3 Table — (DOCX) [file pone.0127157.s004.docx]

**S3 Table.** The correlations between ADHD symptoms and time perception tasks in the absolute discrepancy score/in the accuracy coefficient scores

| Correlations | verbal estimation | Time reproduction  single version | Time reproduction  dual task simple version | Time reproduction  dual task difficult version |
| --- | --- | --- | --- | --- |
| Inattentive | 0.23*/0.23* | 0.12/-0.07 | 0.14/-0.07 | 0.26**/-0.21* |
| Impulsivity/  hyperactivity | 0.09/0.11 | 0.12/-0.12 | -0.02/0.00 | 0.07/-0.17 |

**Note.** **p*<.05, ***p*<.01
