# Supplementary material for: Interval Timing Deficits Assessed by Time Reproduction Dual Tasks as Cognitive Endophenotypes for Attention-Deficit/Hyperactivity Disorder
Source: PLoS One. 2015 May 18;10(5):e0127157. doi: 10.1371/journal.pone.0127157 (PMC4436371; doi:10.1371/journal.pone.0127157)
Supplement: S4 Table — (DOCX) [file pone.0127157.s005.docx]

**S4 Table.** The regression analyses for ADHD symptoms as predictors and time reproduction dual task (difficult) as the dependent variables in the absolute discrepancy score

|  | Time reproduction (dual task difficult) | | |
| --- | --- | --- | --- |
|  | *Β* | *t* | Unique *R^2^* |
| Inattentive | 0.28 | 2.75** | 0.061 |
| Impusivity/  hyperactivity | -0.06 | -0.57 | 0.003 |
| Overall *R^2^* | 0.07 |  |  |

**Note.** **p*<.05, ***p*<.01
