# Supplementary material for: Interval Timing Deficits Assessed by Time Reproduction Dual Tasks as Cognitive Endophenotypes for Attention-Deficit/Hyperactivity Disorder
Source: PLoS One. 2015 May 18;10(5):e0127157. doi: 10.1371/journal.pone.0127157 (PMC4436371; doi:10.1371/journal.pone.0127157)
Supplement: S5 Table — (DOCX) [file pone.0127157.s006.docx]

**S5 Table.** Gender and interval length effect in verbal estimation and time reproduction tasks

| Mean(SD) | male(n=294) | female(n=118) | *F* value^a^ |
| --- | --- | --- | --- |
| Absolute Discrepancy Scores | | | |
| Verbal estimation task | | |  |
| 5 sec | 1.62(1.76) | 1.79(2.18) | Gender: *F*=1.34, *p*=.249 |
| 12 sec | 3.65(4.09) | 4.23(4.90) | IN: *F*=27.63, *p*< .001 |
| 17 sec | 4.86(5.10) | 6.17(7.13) | Gender×IN: *F*=2.61, *p*=.074 |
| Time reproduction (single) | | |  |
| 5 sec | 0.71(0.45) | 0.81(0.53) | Gender: *F*=0.30, *p*= .583 |
| 12 sec | 1.72(1.45) | 1.40(1.21) | IN: *F*=17.86, *p*< 001 |
| 17 sec | 2.25(2.19) | 2.39(2.11) | Gender×IN: *F*=2.67, *p*=.070 |
| Time reproduction (dual task simple version) | | |  |
| 5 sec | 1.09(0.69) | 1.13(0.66) | Gender: *F*=1.02, *p*=.315 |
| 12 sec | 2.75(1.79) | 2.96(2.12) | IN: *F*=28.46, *p*< .001 |
| 17 sec | 4.23(2.67) | 4.68(2.76) | Gender×IN: *F*=0.90, *p*=.406 |
| Time reproduction (dual task difficult version) | | |  |
| 5 sec | 1.33(0.79) | 1.45(0.84) | Gender: *F*=0.06, *p*=.813 |
| 12 sec | 3.36(2.13) | 3.48(2.09) | IN: *F*=66.08, *p*< .001 |
| 17 sec | 5.16(3.30) | 5.41(3.32) | Gender×IN: *F*=0.07, *p*=.931 |
| Accuracy Coefficient Scores | | | |
| Verbal estimation task | | |  |
| 5 sec | 1.26(0.65) | 1.22(0.56) | Gender: *F*=0.64, *p*=.425 |
| 12 sec | 1.19(0.51) | 1.19(0.58) | IN: *F*=1.67, *p*=.189 |
| 17 sec | 1.19(0.56) | 1.19(0.59) | Gender×IN: *F*=0.87, *p*=.421 |
| Time reproduction (single) | | |  |
| 5 sec | 0.91(0.21) | 0.90(0.18) | Gender: *F*=0.03, *p*=.871 |
| 12 sec | 0.88(0.17) | 0.90(0.14) | IN: *F*=17.60, *p*< .001 |
| 17 sec | 0.88(0.17) | 0.87(0.17) | Gender×IN: *F*=1.03, *p*=.358 |
| Time reproduction (dual task simple version) | | |  |
| 5 sec | 0.85(0.25) | 0.82(0.22) | Gender: *F*=2.37, *p*=.124 |
| 12 sec | 0.80(0.20) | 0.74(0.23) | IN: *F*=15.47, *p*< .001 |
| 17 sec | 0.75(0.19) | 0.73(0.21) | Gender×IN: *F*=0.79, *p*=.455 |
| Time reproduction (dual task difficult version) | | |  |
| 5 sec | 0.79(0.28) | 0.76(0.28) | Gender: *F*=0.30, *p*=.587 |
| 12 sec | 0.73(0.21) | 0.72(0.23) | IN: *F*=20.34, *p*< .001 |
| 17 sec | 0.70(0.21) | 0.67(0.24) | Gender×IN: *F*=0.57, *p*=.564 |

**Note.** M, mean; SD, standard deviation; Gender, gender main effects, IN, lengths of interval main effects;
